# Schwannoma of the Base of the Tongue: A Case Report of a Rare Disease and Review of Literatures

**DOI:** 10.1155/2020/7942062

**Published:** 2020-12-31

**Authors:** Mohd. Yusuf Haider, Manjur Rahim, N. M. K. Bashar, Md. Zakir Hossain, Sk Md Jaynul Islam

**Affiliations:** ^1^Department of ENT, Colonel Malek Medical College, Manikganj, Bangladesh; ^2^Department of ENT, 250 Bed District Hospital, Manikganj, Bangladesh; ^3^Department of Anesthesiology, Colonel Malek Medical College, Manikganj, Bangladesh; ^4^Armed Forces Institute of Pathology, Dhaka, Bangladesh

## Abstract

**Background:**

Schwannoma is a benign nerve sheath tumor. It was first identified by Virchow in 1908. These tumors can emerge from any nerve covered with a Schwann cell sheath, including the cranial nerves (with the exception of the optic and olfactory nerves), the spinal nerves, and the autonomous nervous system (Harada H, Omura K and Maeda A, 2001). *Case Presentation*. A 28-year-old male farmer presented with a swelling at the right side of the base of tongue extending into the oral tongue. It was identified incidentally by his newly married wife while he was yawning. It was asymptomatic. The patient had no difficulty in chewing, swallowing, or phonation and also no sensory or taste abnormalities. The tongue movements were normal.

**Conclusions:**

Diagnosis of schwannoma should be considered for a smooth, painless, firm swelling in the tongue. A schwannoma of the tongue may grow large enough before producing any symptom. Around 25–40% of schwannoma happen within the head and neck region, and among these, 1-12% occurs in the oral cavity, most regularly the tongue or mouth floor. Schwannoma of the tongue does not show any age or sex predisposition. It usually presents as a painless lump in the tongue, but when it grows larger than 3.0 cm, it may produce dysphagia, pain, or discomfort and change in the quality of voice. Here, we report a case of large (4 cm × 3 cm) asymptomatic schwannoma of the tongue in a 28-year-old male patient and review the literature available during the last 61 years.

## 1. Introduction

Schwannoma is a benign nerve sheath tumor. It was first identified by Virchow in 1908. These tumors can emerge from any nerve covered with a Schwann cell sheath, including the cranial nerves (with the exception of the optic and olfactory nerves), the spinal nerves, and the autonomous nervous system [1]. When the nerve of origin is small, it can be difficult to demonstrate its connection with a given tumor. On the other hand, if the site of origin is a larger nerve, it is observed that the nerve fibers are splayed over the outer side of the capsule instead of being absorbed into the tumor mass [2]. About 25–45% of all schwannomas occur in the head and neck [3]. Around 1–12% of these occur intraorally [4, 5] with the tongue being the most common site [5, 6]. Although there are many case reports of tongue schwannomas in the literature, after Hatziotis et al. [6], there has been no comprehensive review of the literature. We present a case of tongue schwannoma and study the literature available from the last 61 years (1959–2019).

## 2. Materials and Methods

A PubMed search for the terms “tongue schwannoma,” “lingual schwannoma,” “tongue neurilemmoma,” and “lingual neurilemmoma” was conducted with the 1959–2019 date range. The search was restricted to English case reports. Unless the ventral tongue was also involved, mouth floor schwannomas were not included. All the case reports had confirmed the masses' identity as schwannomas histologically. From the case reports for data analysis, the following elements were extracted: age, gender, location of schwannoma (anterior one-third vs. posterior two-thirds of tongue), symptoms, tumor size, and treatment modality.

## 3. Case Report

A 28-year-old male farmer presented with a swelling at the right side of the base of tongue extending into the oral tongue. It was identified incidentally by his newly married wife while he was yawning. It was asymptomatic. The patient had no difficulty in chewing, swallowing, or phonation and also no sensory or taste abnormalities. The tongue movements were normal.

**Figure 1:**
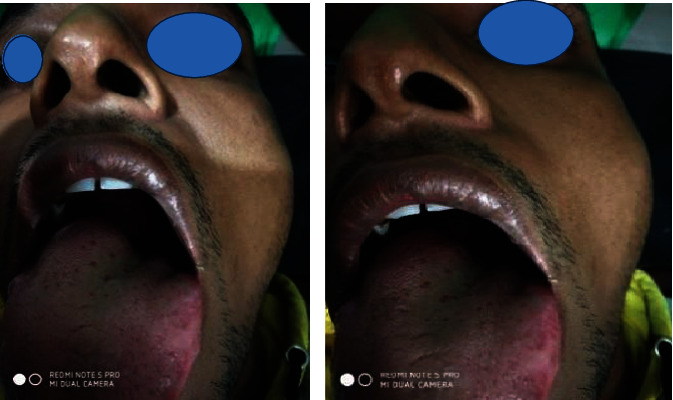
Large asymptomatic swelling at posterolateral aspect of right side of tongue.

**Figure 2:**
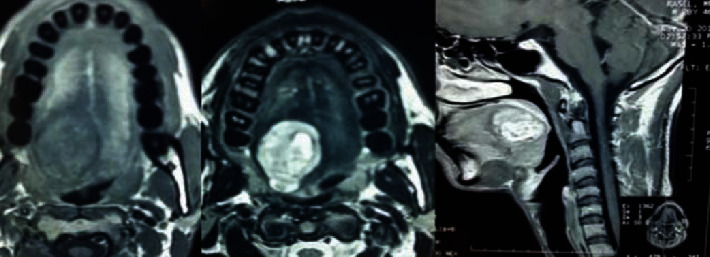
Axial T1 and T2 and sagittal T1-weighted magnetic resonance image showing a well-defined mass centered right to the midline in the base of the tongue. The tumor has a smooth well-defined border, with no invasion.

**Figure 3:**
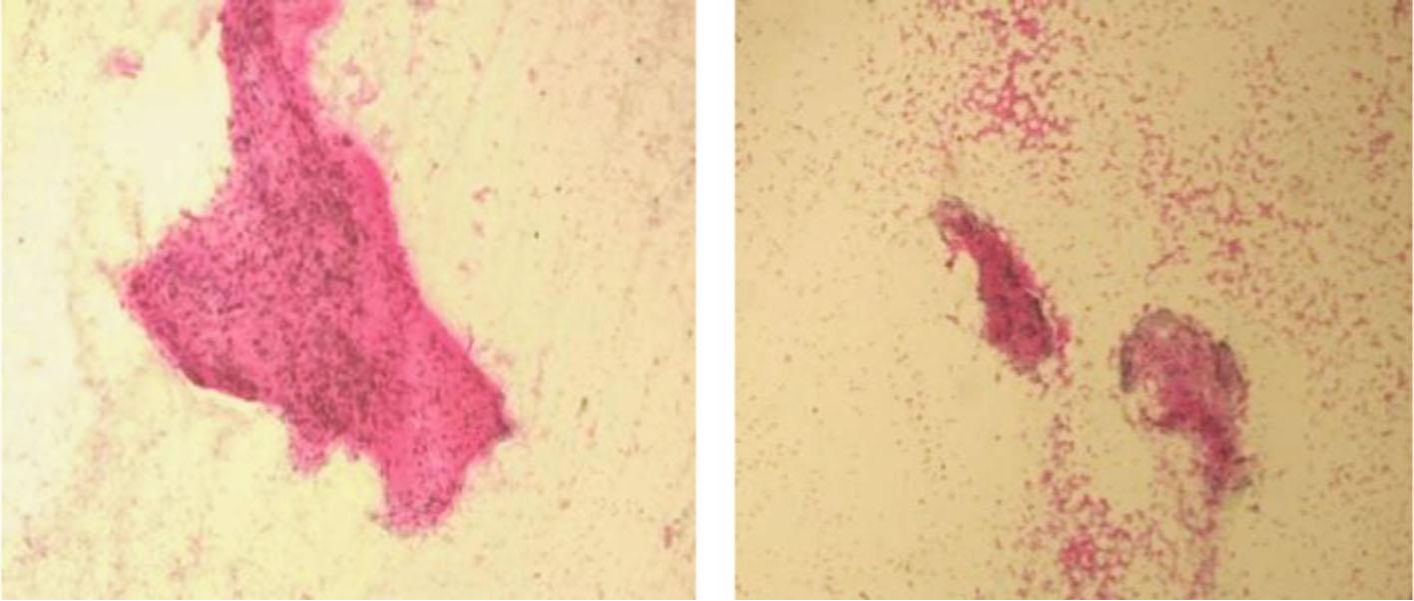
FNAC: fragment-of-spindle-cells.

**Figure 4:**
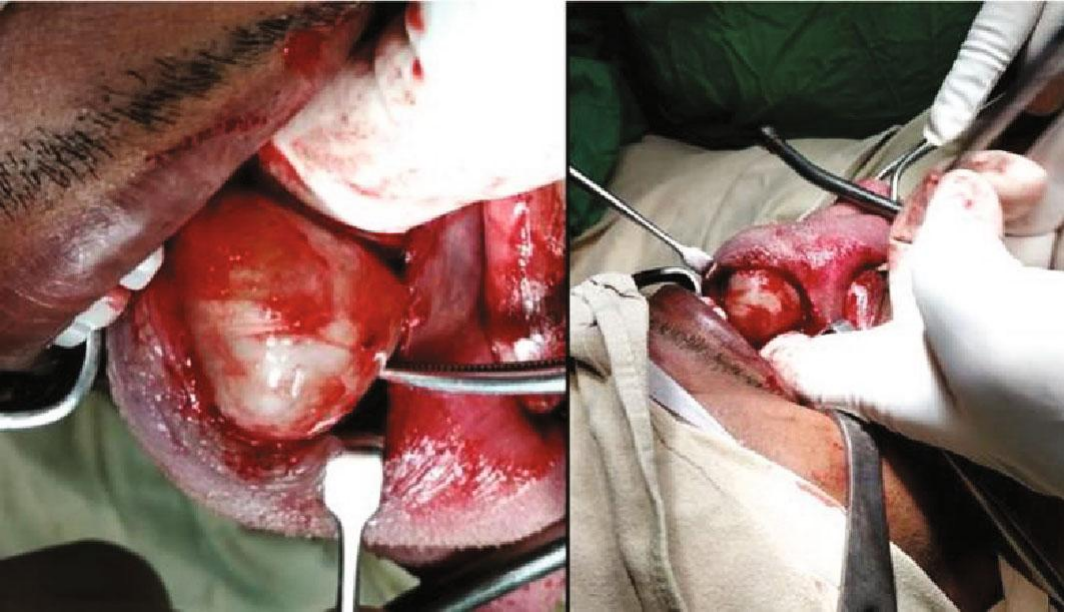
Tumor is being removed from the base of the tongue.

**Figure 5:**
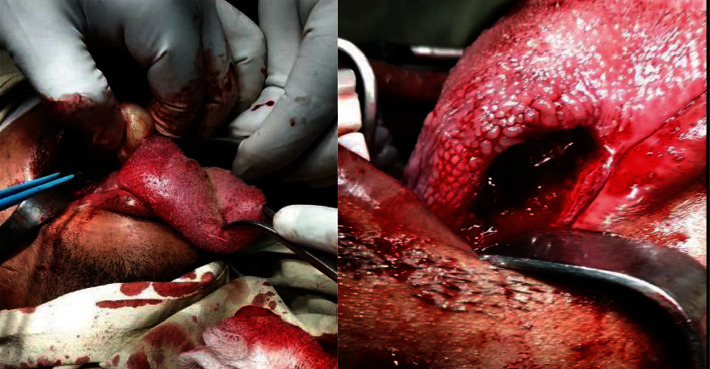
Surgical field after removal of the tumour.

**Figure 6:**
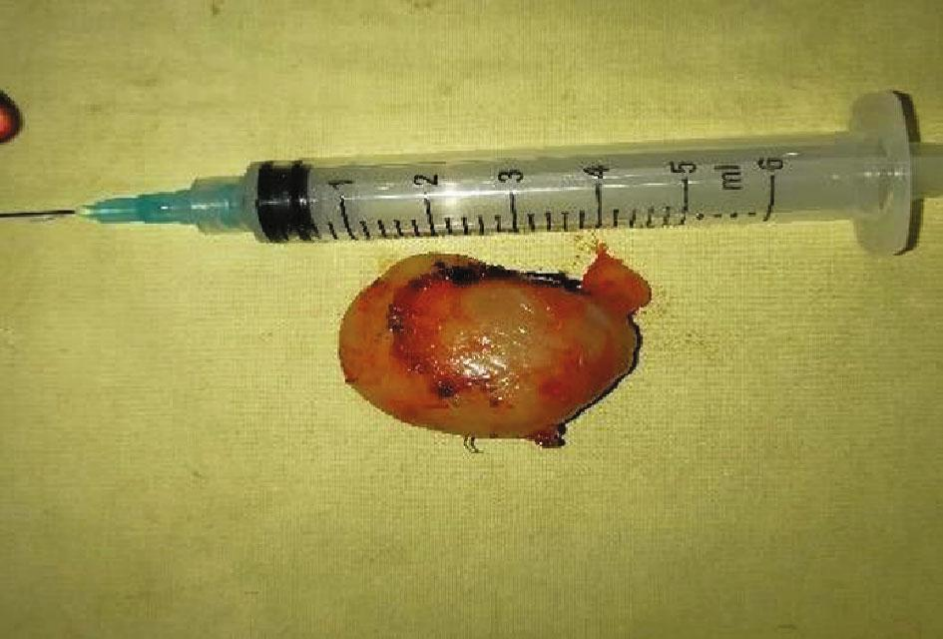
Excised tumour from the base of the tongue.

**Figure 7:**
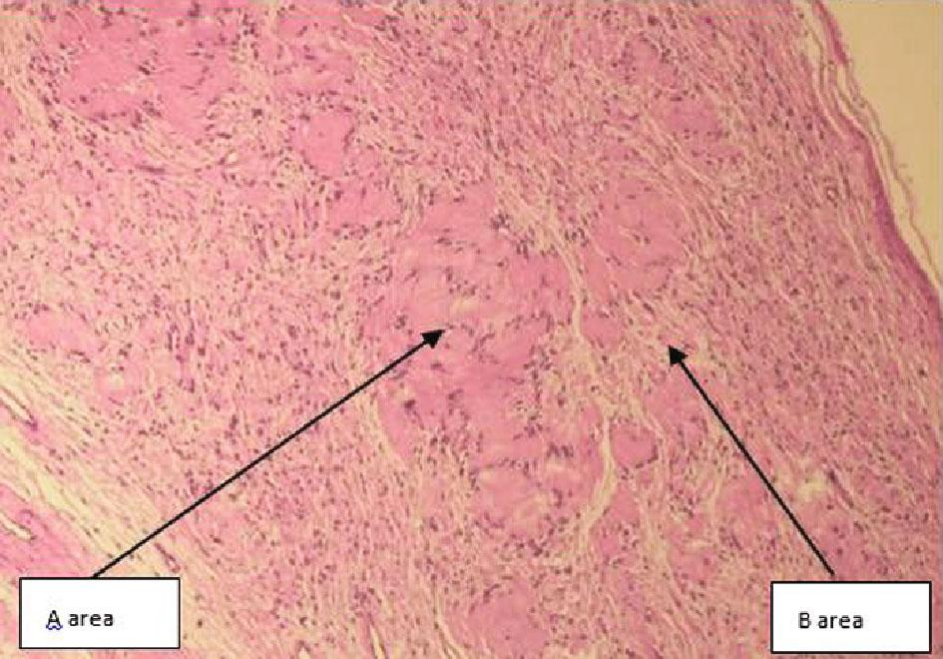
Histopathological examination. The lesion is composed of spindled cells with hypercellular Antoni A areas and hypocellular Antoni B areas.

**Figure 8:**
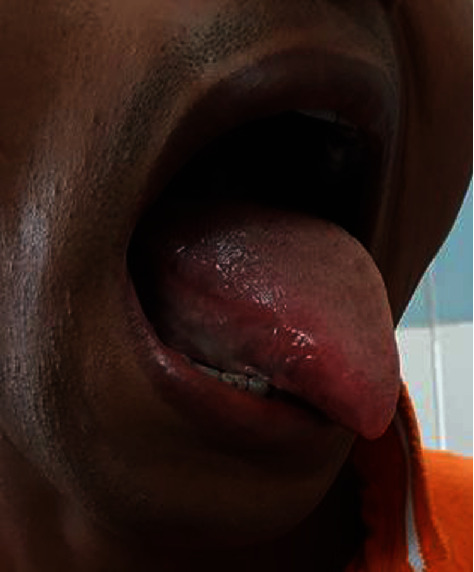
The patient followed up after 1 (one) year, and there was no recurrence of the disease.

On examination, there was an oval swelling at the right side of base of the tongue measuring about 4 cm × 3 cm (Figure 1). The surface was smooth, margin regular, and no discoloration or distortion of tongue epithelium. It was nontender, farm in consistency, and was not fixed with underlying or overlying structures. The remaining oral cavity examination was normal; nasopharyngolaryngoscopy revealed no abnormality in the adjacent areas. There was no cervical lymph node enlargement. Clinically, it appeared like a dermoid cyst or lipoma. MRI of the tongue manifested hyperintense well-circumscribed soft tissue mass in the right half of the base of the tongue on T1/T2-weighted image (Figure 2). It was evaluated with FNAC which revealed benign mesenchymal spindle cell neoplasm, suggestive of nerve sheath tumor with possibility of schwannoma (Figure 3). The patient underwent transoral total excision of the mass under general anesthesia with nasotracheal intubation. For the proper visualization of the base of tongue, frenulum of tongue was incised; tongue was released from floor of mouth and pulled out. An incision was given in right lateral margin over the swelling. After splitting the mucosa, mass is exposed, mobilized by blunt dissection, and excised totally (Figures 4–6). Haemostasis was ensured, and wound closed in layers. Histopathological report revealed features of schwannoma (Figure 7). For confirmation of the tissue of origin, immunocytochemistry was done and found strongly positive for S100 protein. There was no symptom or sign of recurrence in 12 months postoperative follow-up (Figure 8).

## 4. Discussion

Though this is not clear of the etiology of the schwannoma, it is known to be derived from nerve sheath Schwann cells, which surround cranial, peripheral, and autonomic nerves [6, 7]. The head and neck are rather common location of this neoplasm. Intraoral schwannoma mainly arise from the ongue, followed by the palate, mouth floor, buccal mucosa, gingiva, lip, and vestibule [8, 9], though the tongue is most commonly involved [10]. The lesion is slow growing, and thus, its onset is usually long before presentation. Lingual schwannoma shows no age or gender predisposition [11]. Usually, it presents as a painless lump in any part of the tongue. The average size at presentation was 2.4 cm. However, when the mass exceeds 3.0 cm, dysphagia, pain (or discomfort), dysphonia, and voice changes are usually present (Table 1).

In the literature review of 61-year period (from 1959 to 2019), 68 cases schwannoma of the tongue were found, and 54% of them are male, and the rest of them are female. More than half of the cases were posterior tongue schwannomas (56%). According to this review, the patients had feeling of lump cases, respectively.

The mean age at diagnosis was nearly 25 years. Transoral excision was performed in 96% cases. However, for two cases, carbon dioxide laser was used for the tongue-base schwannoma, and in three cases, submandibular approach was used. There was no report of recurrence.

**Figure 9:**
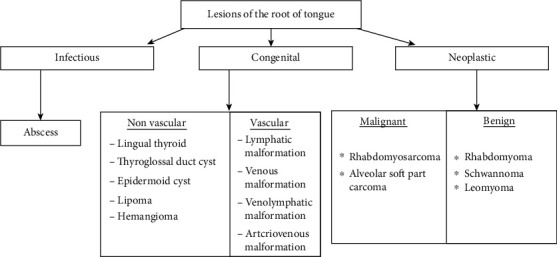
Base of the tongue lesions can be subcategorized as above.

Clinically, the schwannomas may be indistinguishable from other encapsulated benign tumors, because biopsy and histological examination are essential to formulate a correct diagnosis. An excisional biopsy was performed to formulate a correct diagnosis and finally find out that the case was uncomplicated. Imaging has become an integral part of evaluation for tongue base lesions, and thus, a systematic imaging approach should be considered. As demonstrated in Fig.  9, lesions of the tongue can be divided into infectious, neoplastic, and congenital categories. An infectious process, such as an abscess, appears hyperintense on T2WI with a thick rimenhancing margin [12]. The present case was totally asymptomatic before surgery, and there was no major complication of surgery. The option of complete resection was chosen on the basis of the size of the lesion and the age of the patient.

## 5. Conclusions

Diagnosis of schwannoma should be considered for a smooth, painless, firm swelling in the tongue. A schwannoma of the tongue may grow large enough before producing any symptom. Total excision is the treatment of choice particularly in a young patient as it continues to grow. Most of the cases can be completely excised transorally. Total surgical excision of the lesion does not result in any recurrence.

## Figures and Tables

**Table 1:** Patients and tumor characteristics of tongue schwannomas.

Author	Year	Gender	Age	Size (cm)	Site	Presentation	Surgical approach
Mercantini and Mopper [13]	1959	M	22	1	Anterior	Intermittent pain	Transoral
Cameron [14]	1959	M	25	1.5	Anterior	Lump	Transoral
Chadwick [15]	1964	F	20	2.2	Posterior	Lump	Transoral
Craig [16]	1964	F	8	3	Posterior	Lump	Transoral
Pantazopoulos [17]	1965	F	45	4.5	Posterior	Dysphasia/change in voice	Transoral
Chamber [18]	1965	M	29	5	Posterior	Throat discomfort	Transoral
Fifer et al. [19]	1966	F	28	3	Anterior	Lump	Transoral
Hatziotis and Aspride [20]	1967	M	25	Hazelnut	Posterior	Lump	Transoral
Oles and Werthemier [21]	1967	M	52	1	Anterior	Lump	Transoral
Paliwal et al. [22]	1967	M	32	2.5	Anterior	Lump	Transoral
Crawford et al. [23]	1968	M	23	0.5	Anterior	Lump	Transoral
	1968	M	24	1	Anterior	Lump	Transoral
Das Gupta et al. [24]	1969	F	21	5	Posterior	Pain	Transoral
Bitici [25]	1969	M	40	2.5	Anterior	Slight discomfort	Transoral
Sinha and Samuel [26]	1971	M	23	1.5	Posterior	Dysphagia	Transoral
Mosadomi [27]	1975	M	19	3	Anterior	Painful mass	Transoral
Swangsilpa et al. [28]	1976	M	26	3	Anterior	Lump	Transoral
Sharan and Akhtar [29]	1978	F	30	1.5	Anterior	Change in voice	Transoral
Akimoto et al. [30]	1987	M	15	1	Anterior	Lump	Transoral
Sira et al. [31]	1988	F	18	3	Posterior	Lump	Transoral
Flickinger et al. [32]	1989	F	28	3	Anterior	Lump	Transoral
Talmi et al. [33]	1991	F	75	1	Posterior	Lump	Transoral
Gallesio and Berrone [34]	1992	F	21	1.9	Anterior/base	Dysphonia/paresthesia/chewing difficulty	Transoral
Lopez and Ballistin [10]	1993	M	24	0.6	Anterior	Lump	Transoral
Haring [35]	1994	F	49	2	Anterior	Lump	Transoral
Nakayama et al. [36]	1996	F	40	5.5	Anterior	Lump	Transoral
Dreher et al. [37]	1997	F	31	3	Base	Dysphagia	Transoral
Spandow et al. [38]	1999	M	37	7.9	Posterior	Throat discomfort	Transoral
de Bree et al. [2]	2000	F	24	5	Posterolateral/base	Lump	Submandibular
Pfeifle et al. [39]	2001	F	30	0.3	Anterior	Lump	Transoral
Cinar et al. [40]	2004	M	7	1	Anterior	Lump	Transoral
Bassichis and McMlay [41]	2004	M	9	2.3	Posterior/base	Snoring	Transoral
Nakasato et al. [42]	2005	F	9	2	Posterolateral/base	Bleeding/ulceration	Transoral
Hwang et al. [43]	2005	M	23	2.8	Anterior	Lump	Transoral
Lopez-Jornet and Bermejo-Fenoll [44]	2005	M	39	0.8	Posterolateral/base	Lump	Transoral
Vafiadis et al. [45]	2005	M	18	3.1	Anterior	Lump	Transoral
Bansal et al. [46]	2005	M	26	4	Posterolateral/ventral	Paresthesia/dysphonia	Transoral
Hsu et al. [7]	2006	M	20	5	Posterior/base	Bleeding	Transoral
Ying et al. [47]	2006	F	26	4	Posterior/base	Dysphagia/otalgia	Transoral
Enoz et al. [48]	2006	M	7	2.5	Anterior/base	Dysphagia/pain	Transoral
Mehrzad et al. [49]	2006	M	49	2.2	Posterior/ventral	Pain	CO2-transoral
Batra et al. [50]	2007	M	30	3	Posterolateral/base	Dysphagia, dyspnea, abscess	Transoral
Ballesteros et al. [51]	2007	F	31	2	Base	Pain	CO_2_-transoral
Sawhney et al. [52]	2008	F	37	4.6	Posterolateral/base	Dysphagia/snoring	Submandibular
Sethi et al. [53]	2008	F	28	1	Anterolateral/ventral	Lump	Transoral
Pereira et al. [54]	2008	M	12	1.5	Posterolateral/ventral	Lump	
Cohen and Wang [55]	2009	M	77	0.7	Posterolateral/ventral	Lump	Transoral
Gupta et al. [56]	2009	F	18	1	Anterior/ventral	Lump	Transoral
Mardanpour and Rahbar [57]	2009	M	18	2	Posterior	Dysphagia/change of voice	Transoral
Karaca et al. [58]	2010	F	13	2	Posterolateral/ventral	Dysphagia	Transoral
Cigdem et al. [59]	2010	M	13	2	Anterior/ventral	Lump	Transoral
Jeffcoat et al. [60]	2010	M	68	1.5	Lateral	Lump	Transoral
Naidu and Sinha [61]	2010	M	12	2	Anterolateral/base	Paresthesia/bleeding/ulceration	Transoral
Lukšić et al. [62]	2011	M	10	1.5	Posterolateral/ventral	Lump	Transoral
Batra et al. [63]	2011	F	38	4.2	Posterior/ventral	Dysphagia/change of voice	Transoral
Nisa et al. [64]	2011	F	38	8.5	Posterolateral/ventral	Dysphagia/dysphonia/dyspnea	Transoral
Monga et al. [65]	2013	M	20	2	Posterolateral/base	Lump	Transoral
Lira et al. [5]	2013	F	26	2.5	Posterior/ventral	Cervical pain	Transoral
Erkul et al. [66]	2013	M	21	3	Posterolateral/ventral	Chewing difficulty	Transoral
Jayaraman et al. [67]	2013	F	25	3	Anterolate	Lump	Transoral
George et al. [4]	2014	M	26	4	Posterolateral/base	Dysphagia/dysphonia	Transoral
Bhola et al. [11]	2014	F	14	1.5	Anterolateral/ventral	Lump	Transoral
Moreno-Garcíaet al. [68]	2014	F	13	2	Anterior/ventral	Lump	Lipsplit/mandibulotomy
Nibhoria et al. [69]	2015	F	18	1.5	Posterolateral/ventral	Lump	Transoral
Gopalakrishnanetal. [70]	2016	M	32	3	Posterolateral/ventral	Dysphagia	Transoral
Sharma and Rai [71]	2016	F	20	4	Posterolateral/ventral	Dysphagia/dysphonia	Transoral
Kavčič and Božič [72]	2016	F	20	1.3	Anterolateral/ventral/tip	Lump	Transoral
Lee et al. [73]	2016	M	28	4	Posterior/ventral	Lump	Transoral
Zain et al. [12]	2016	F	24	Not clear	Posterior	Lump	Transoral
Steffi Sharmaetal.	2018	F	20	4	Posterior	Lump	Transoral
Current	2019	M	28	4	Posterior	Lump	Transoral
